# Socioeconomic disadvantage and oral-health-related hospital admissions: a 10-year analysis

**DOI:** 10.1038/bdjopen.2016.4

**Published:** 2016-07-29

**Authors:** Estie Kruger, Marc Tennant

**Affiliations:** 1 International Research Collaborative - Oral Health and Equity, School of Anatomy, Physiology and Human Biology, Faculty of Sciences, The University of Western Australia, Nedlands, WA, Australia

## Abstract

**Objective:**

The aim of this Western Australian population study was to assess the relationship of socioeconomic disadvantage and: 1) trends in hospitalisations for oral-health-related conditions over 10 years; 2) insurance status, costs and length of stay in hospital; and 3) specific conditions (principal diagnosis) patients were admitted for.

**Methods:**

Hospitalisation data (of oral-health-related conditions) were obtained for every episode of discharge from all hospitals in Western Australia for the financial years 1999–2000 to 2008–2009. Area based measures (using the Index of Relative Socioeconomic Disadvantage) was used to determine relationships between socioeconomic status and other variables.

**Results:**

The most disadvantaged in the population are being hospitalised at significantly higher rates than other groups, stay in hospital for longer, and at higher costs. This trend remained over a period of 10 years. Those least disadvantaged have the second highest rates of hospitalisation, but the likelihood of being admitted for different procedures differ between these two extremes.

**Conclusions:**

The importance of socioeconomic determinants of health are evident when analysing these hospitalisations. Recognition that lifestyle choices are severely restricted among the most marginalised and disadvantaged groups in the population can no longer be ignored in attempts to reduce health inequalities.

## Introduction

The social gradient in health means that health inequities affect all, and the poorest of the poor have the worst health. This is a global phenomenon, and is seen in low, middle and high income countries.^1^ It is social and economic conditions, and their effects on people’s lives, that determine their risk of illness and the actions taken to prevent them becoming ill or treat illness when it occurs.^1^ The mechanisms by which socioeconomic status (SES) influence health status are complex and varied, and this association is confounded by many factors. It is hypothesised that a number of inter-related factors including education, place of residence, health beliefs and behaviour, occupation, income, access to health services and the environment in which people live determine the socioeconomic disadvantage and health.^2^ This relationship exists across a very broad range of health indicators, including dental health.^3^

Strong evidence exists for the relationship between oral health and socioeconomic status in the Australian population.^4–13^ Many studies have focused on child oral health, but inequities also exists in the adult population. In the Australian dentate population, adults with lower levels of household income and educational attainment suffered greater tooth loss, greater social impact of oral conditions on quality of life and worse subjective oral health.^4,5^

Australia has a complex health system, and the provision of oral health care depend on a combination of private and public providers and funders. Differences in access to care (among other factors) inevitably result in inequities in health, and this is no more evident than when comparing different socioeconomic groups in the population. Barriers to better public oral-health outcomes for socially disadvantaged Australians include service rationing of oral health care and marginalisation of oral health in policy and funding. Dental services are one of the least subsidised areas of health.^14^

Although studies of health inequalities are carried out worldwide, the development and increasing use of new measures of socioeconomic status have improved this area of research. These measures employ the use of census data on small areas to classify individuals in terms of the level of material deprivation in the area in which they live.^3,6^ Use of these area-based indices are based on assumptions that aggregate community-level variables are important explanatory factors in health outcomes above and beyond individual level circumstances.^3,6,15^ Ecological factors can be seen as upstream determinants of health and disease status in a population, and there is a growing awareness of the impact of neighbourhood factors on individual health outcomes.^6^ An Australian study confirmed that the socioeconomic characteristics of neighbourhoods are important for oral health over and above the socioeconomic characteristics of the people living in those neighbourhoods.^16^

Previous work indicated that adult hospitalisations for oral-health-related conditions remain considerable, even though a large proportion might be preventable.^11–13^ According to the social gradient theory it would be expected that those who suffer poorer oral health, would be hospitalised at higher rates, and that hospital admissions for treatment of oral-health-related conditions should be associated with the burdens of disease within the population. The aim of this Western Australian population study was to assess the relationship of socioeconomic disadvantage (using area-based measures) and:

1. Trends in hospitalisations for oral-health conditions over 10 years,
2. Insurance status, costs and length of stay in hospital, and
3. Specific conditions (principal diagnosis) patients were admitted for.

## Materials and methods

### Ethics

Ethics approval for this study was obtained from the Human Research Ethics Committee at the University of Western Australia, reference number RA/4/1/5502.

### Study population

This included all adults in WA who were admitted to hospital for an oral-health-related condition, over a 10-year period. The adult population in WA (all 18 years and older), were 1,059,750 in 1999, 1,094,197 in 2001 and 1,221,799 in 2006.^17^

### Hospitalisation data

Hospitalisation data were obtained from the Western Australian Morbidity Data System. The principal diagnosis, as classified by the International Classification of Disease (ICD-10AM),^18^ was obtained for every episode of discharge from all private and public hospitals in Western Australia for the financial years 1999–2000 to 2008–2009. In this study hospitalisation episodes were selected on the basis of a principal diagnosis (the primary condition under treatment) being an oral-health-related condition.

### Population rates and cost

Population data for rate calculations were obtained from the estimates as calculated by the Western Australian Department of Health. These estimates were extrapolated from census data collected by the Australian Bureau of Statistics. Estimated cost of care was determined for each episode using the national standard diagnostic-related group (DRG) average price. The Australian Refined Diagnosis Related Group (AR-DRG), version 5.1, National Centre for Classification in Health (NCCH), Sydney, NSW, Australia was used to calculate the direct cost. AR-DRG is an Australian admitted patient classification system, which provides a clinically meaningful way of relating the number and type of patients treated in a hospital to the resources required by the hospital. Each AR-DRG represents a class of patients with similar clinical conditions requiring similar hospital services.^19^

### Socioeconomic status

The Socioeconomic Indexes for Areas (SEIFA) is a widely used measure of geographically concentrated disadvantage. SEIFA was created by the Australian Bureau of Statistics who broadly define relative socioeconomic advantage and disadvantage in terms of people’s access to material and social resources, and the ability to participate in society.^20^ SEIFA is composed of four indexes, namely: the Index of Relative Socioeconomic Disadvantage (IRSD); the Index of Relative Socioeconomic Advantage and Disadvantage; the Index of Economic Resources; and the Index of Education and Occupation. In this study the IRSD was used as the area-based composite measure of SES, and this index is derived from variables as indicated in Table 1.^20^ The IRSD score of the residential statistical local area of each person admitted to hospital, was used a measure of socioeconomic status.

### Statistical analysis

All rates were calculated using the Rates Calculator (Perth, WA, Australia), a software package developed by the WA Department of Health. All rates were calculated per 100,000 person years, and were adjusted for ages and IRSD quintile. Significant differences between rates were based on non-overlapping 95% confidence intervals (*P*<0.05). Means between groups were compared using analysis of variance. Odds ratios and confidence intervals were calculated using logistic regression for the increased likelihood of being hospitalised for each of the specific categories of principal diagnosis (according to ICD-10 Code for each admission). All statistical analysis were undertaken using IBM SPSS Statistics 19 (IBM, New York, NY, USA).

## Results

### Demographics

Over a 10-year period, a total of 131,509 people were admitted to hospitals in WA for oral-health-related conditions. Slightly more females (51.7%) were admitted (Table 2). The majority of those hospitalised (97%) were non-Indigenous persons, and between the ages of 18 and 39 years (63%; Table 2). Only 2% were above the age of 80 years. Over the 10-year period, there was an increase in the numbers hospitalised for every year. Almost two-thirds (63%) of patients admitted to hospital has private insurance (Table 1). Almost half (47%) of all those admitted were from areas classified as IRSD quintile 5 (least disadvantaged), and 6.5% were from the most disadvantaged areas (IRSD quintile 1).

### Principal diagnosis

Almost half (49%) of all hospitalisations was for the removal of ‘Embedded and/or impacted teeth’. ‘Dental caries’ accounted for almost one-tenth of all admissions (9%) and 8.5% were admitted for ‘Other disorders of teeth and supporting structures’ (Table 1). The 10 most common conditions for which people were admitted were the following (conditions as categorised according to ICD code): ‘Embedded and Impacted teeth’; ‘Dental Caries’; ‘Other disorders of teeth and supporting structures’; ‘Other Fractures’ (which include fractures of teeth, palate, nasal bone, alveolus, lower facial bones); ‘Malignant neoplasms’; ‘Pulp and periapical conditions’; ‘Other diseases of the jaw’; ‘Jaw fractures’ (maxilla and mandible); ‘Dento-facial anomalies’; and ‘Gingivitis and Periodontitis’.

### Trends over time

Rates were calculated for the overall 10-year period and found that the average rate over the study period were highest for those in the most disadvantaged areas. The second highest average rate were for those from the least disadvantaged areas (Table 3). The rate for quintile 1 was significantly higher than any of the others (*P*<0.05), the rate for quintile 5 also differed significantly from all the others (*P*<0.05), and the rate for quintile 3 was significantly lower that any of the others (*P*<0.05; Table 3).

**Figure 1:**
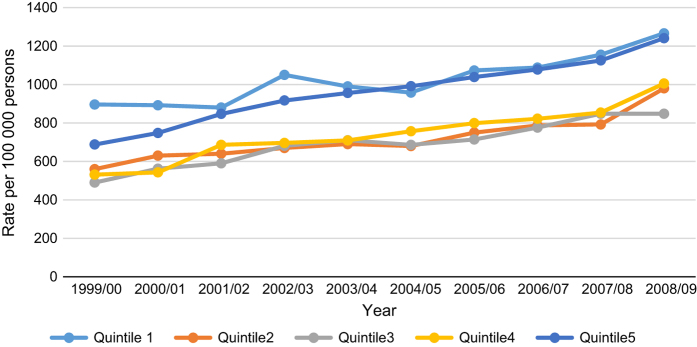
Rates of hospitalisation over 10 years by IRSD quintile.

**Figure 2:**
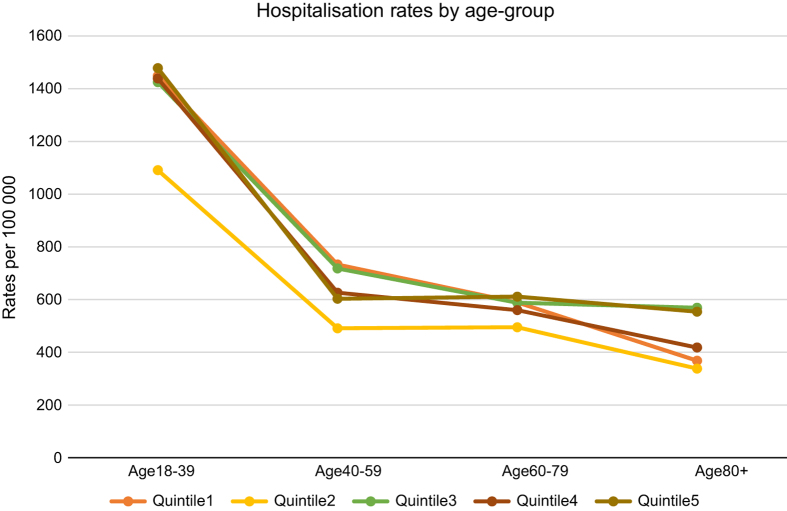
Hospitalisation rates by age group and relative socioeconomic disadvantage.

Over time, rates were increasing for all socioeconomic groups, and the highest rates for each year remained for those from the most disadvantaged areas. Lowest rates for each year were for those in the third quintile (Figure 1). The rates across all years for the most disadvantaged groups remained significantly higher than any of the others groups (*P*<0.05). Hospitalisation rates by age-group indicated that those in the youngest age category (18 to 39 years) consistently had the highest rates of hospitalisation, across all IRSD quintiles. It was the highest however, for those from the most disadvantaged quintile. Rates decreased by age across all the socioeconomic groups, and was lowest for those in the oldest (80 years+) age category (Figure 2). Except for the third quintile, rates for the youngest age category in all other SES groups, were significantly higher (*P*<0.05) than the other age categories within the same SES group (Figure 2).

### Socioeconomic status and length of stay, cost and insurance status

There was an increase in the proportions of those patients with private health insurance across the SES groups, from the lowest (28.5%) in the most disadvantaged group, to the highest (74.6%) of those in the least disadvantaged group (Table 4). Those from the poorest quintile stayed, on average, in hospital the longest (2.07 days), as opposed to those from the richest quintile, who stayed, on average, the shortest (1.37 days). On average the mean direct costs (DRG costs) per hospitalisation episode were highest for those from the poorest group (AU$3642), and lowest for those from the richest group (AU$2942; Table 4).

### Socioeconomic status and principal diagnosis

Deprivation of area of residence was found to be associated with the principal diagnosis (condition for which hospitalisation was required). There was a statistically significant trend for those living in the most disadvantaged areas to be at higher risk for hospitalisation for most conditions (Table 5). The relationship was reversed, however, for admission to hospital for the removal of embedded and impacted teeth. In the youngest age category those from the most disadvantaged areas were 76% less likely to be admitted for the removal of embedded and impacted teeth than those from the least disadvantaged area. The same trend were seen in the other age groups (63% less likely among 40–59-year olds, 58% less likely among 60–79-year olds and 65% less likely among those over the age of 80 years; Table 5).

Among all hospitalised patients, those in the youngest age group and from the most disadvantaged areas were (compared with those from the least disadvantaged areas) almost three times more likely to be admitted for dental caries, almost five times more likely to be admitted for jaw fractures, more than three times more likely to be admitted for malignancies, almost five times more likely to be admitted for other fractures, and more than five times more likely to be admitted for pulp and periapical conditions (Table 5).

The ratios becomes smaller in the older age groups, but for some conditions were still significant. In the age group 40 to 59 years those from the most disadvantaged areas compared with those from the least disadvantaged areas were almost four times more likely to be admitted for jaw fractures, more than twice as likely to be admitted for malignancies, three times more likely to be admitted for other fractures and almost twice as likely to be admitted for pulp and periapical conditions. Those in age groups 60–79 and 80+ years from the most disadvantaged areas were also 3.3 times and almost 3.5 times, respectively, more likely to be admitted for malignancies than those from the least disadvantaged areas (Table 5).

## Discussion

The results of this study indicated consistently higher rates of hospitalisation for oral-health-related conditions among the most disadvantaged group in the WA population, compared with the rest. This trend remained consistent over a period of 10 years, and also remained consistent when analysed by age group, with the youngest, most disadvantaged having significantly higher admission rates than those from other age groups and disadvantage levels. This finding clearly reflects the poorer oral health of groups in the population at the lower end of the socioeconomic scale. Numerous studies have demonstrated this social gradient, not just in Australia^4,21–23^ but it is a worldwide phenomenon.^3^ The study results also indicate that for many, poor oral health ultimately result in hospital admissions, meaning that the condition is not possible to be managed in the primary care system.

Overall, in terms of absolute numbers, 63% of all those admitted had private insurance. When comparing socioeconomic groups, however, 75% of those in the least disadvantaged group had insurance compared with only 28% of those in the most disadvantaged group. In 2010, 55% of Australians had private dental insurance.^23^ Levels of insurance coverage increased across household income, with highest levels of insurance among those with the highest household incomes. For those earning less than $30,000 per year, <30% had private dental insurance.^23^

The importance of private insurance need to be considered against the backdrop of the Australian health-care system, and especially the dental health-care system. Medicare is the basis of Australia's health-care system and covers many health-care costs, but does not cover dental examinations and dental treatment. Australians can choose to have Medicare cover only, or a combination of Medicare and private health insurance. This situation leaves a large part of the population having to pay for dental care, either out-of-pocket, or via private health insurance and those that are less likely to be able to afford private health insurance are those from the most disadvantaged proportions of the population. A safety net exists for the most disadvantaged in the form of access to public dental services, but this does not always include the working poor, who are not eligible for public dental care. Disadvantaged groups that are not eligible for public dental services may have difficulty accessing regular private oral-health services due to the cost, whereas those eligible for public dental care may face long waiting times for care.^14^

The results of this study also indicated that those who are most disadvantaged stayed on average longer in hospital than others, and the average cost per admission was highest in this group. It is estimated that these direct costs (DRG) are very conservative estimations, and in reality, the true costs could amount to double the estimated cost at the patient level (inclusive of health insurance refund). In addition, indirect costs (travel, time off work, support family time and so on) are not included, but others have estimated in small countries with minimal travel that this can be nearly double the direct costs.^24^ Our results thus indicate that those who can least afford it, might have higher costs (direct and indirect) and longer hospital stays.

The condition that most people were admitted to hospital for was for ‘Embedded and Impacted Teeth’. Almost half (48.9%) of all hospitalisations was for this condition. Previous work indicated that these high numbers are driven by the removals of third molars, mostly in younger people.^25–27^ These rates of hospitalisation are much higher in Australia than in some other countries.^26^ This was one of only two conditions where those who are least disadvantaged were significantly less likely to be admitted, and this was seen in all age groups. Those in the youngest and most disadvantaged group were 76% less likely than the youngest and least disadvantaged group to be admitted for this condition, and the same trend was evident in all the other age groups.

The other condition were likelihood to be admitted were significantly less for the poorest compared with the richest, across all age groups was for ‘Other disorders of teeth and supporting structures’. This was especially evident among the older ages (poorest were 84% and 83% less likely than richest in ages 60–79 years and 80+years, respectively).

Calculation of odds ratios for the other most common conditions, especially in the youngest age group, all indicated significantly higher likelihoods of admission (of the most disadvantaged) for each specific condition: this group was almost 5 and 4.8 times more likely to be admitted for ‘Jaw fractures’ and ‘Other fractures’, respectively. Previous work indicated much higher levels of jaw and other fractures among lower socioeconomic groups. The reasons for this include the determinants and risk factors for maxillofacial fractures, which are strongly associated with poverty.^28–31^

The youngest and poorest were 2.6 times more likely to be admitted for ‘Dental caries’, and more than five times more likely to be admitted for ‘Pulp and periapical conditions’ than the youngest least disadvantaged. Previous studies and surveys have emphasised the higher levels of dental caries in Australia among those who are lower on the socioeconomic scale.^4,14^ Admissions for pulp and periapical conditions has previously been shown to be significantly higher in children from poorer socioeconomic backgrounds.^32,33^ Pulp and periapical conditions could result from infections in the tooth, most often caused by untreated dental caries.^34^

For some conditions the trend was evident across all age groups, and the poorest in all age groups were more likely to be hospitalised. ‘Malignancies’ was one such condition. The likelihood was three times more likely among 18–39-year olds, twice as likely in 40–59-year olds, 3.3 times more likely among 60–79-year olds and 3.5 times more likely in those older than 80+ years to be admitted for malignancies than similar age groups in the least deprived group. This might be a reflection of oral cancer being a strongly age-related condition.^35–37^

One weakness of a population-based hospitalisation study like this, is that it cannot determine the need for care, it is unknown whether the care is distributed according to need. However, there are very strong evidence that those who are socioeconomically disadvantaged has higher levels of dental disease. Most population-based studies of this nature rely on indirect inference to evidence relating to disease levels and burdens of disease among different groups. The results of this study thus suggest large and diverging health-care needs between socioeconomic groups.

Access to care is a complicated issue affected by demand and supply barriers that may influence the use of primary health services. The use of primary care services in oral health (where timely and adequate services can be provided), might contribute to less people being hospitalised for treatment of some, but not all conditions. The use of primary dental care services is however not an easy option for all, with multiple factors determining access, including socioeconomic status, geographical location, age and health insurance status, among others.^4,14^ Public dental services in Australia is currently not in a position to provide services to all those who need or demand it^38^ and the results of this study might be a reflection of that.

### Conclusion

The results of this study indicate that the most disadvantaged in the population are being hospitalised for oral-health-related conditions at significantly higher rates than other groups. Those least disadvantaged have the second highest rates of hospitalisation, but the likelihood of being admitted for specific procedures differ between these two extremes. The influence of socioeconomic determinants of health are evident when analysing these hospitalisations. Although the importance of social determinants in oral health is now widely acknowledged, public policy seems to still be focused largely on individual behaviour. Recognition, however, that lifestyle choices are severely restricted among the most marginalised and disadvantaged groups in the population can no longer be ignored in attempts to reduce health inequalities.

## Figures and Tables

**Table 1:** Variables contributing to the Index of Relative Socioeconomic Disadvantage (IRSD)

% Occupied private dwellings with no internet connection
% People age 15 years and over with no post-school qualifications
% People with stated annual household equivalised income between $13,000 and $20,799
% Households renting from Government or community organisations
% People (in the labour force) unemployed
% One parent families with dependent offspring only
% Households paying rent less that $120 per week (excluding $0 per week)
% People aged under 70 who have a long-term health condition or disability and need assistance with core activities
% Occupied private dwellings with no car
% People who identified themselves as being Aboriginal and/or Torres Strait Islander origin
% Occupied private dwellings requiring one or more extra bedrooms
% People aged 15 years and over who are separated or divorced
% Employed people classified as Machinery Operators and Drivers
% People aged 15 years and over who did not go to school
% Employed people classified as Low Skill Community and Personal Service Workers
% People who do not speak English well

**Table 2:** Characteristics of all adult oral-health-related hospital admissions over 10 years in WA

*Variable:*	N *(%)*
*Gender*	
Male	63,569 (48.3)
Female	67,940 (51.7)
All	131,509 (100%)
	
*Indigenous status*	
Indigenous	3,652 (2.8%)
Non-indigenous	12,7857 (97.2%)
All	13,1509 (100%)
	
*Age groups*	
18–39	82,682 (62.9%)
40–59	31,927 (24.3%)
60–79	14,221 (10.8%)
80+	2,679 (2%)
All	131,509 (100%)
	
*Year*	
1999/2000	9,661 (7.3%)
2000/2001	10,415 (8%)
2001/2002	11,693 (8.9%)
2002/2003	12,600 (9.5%)
2003/2004	12,924 (9.8%)
2004/2005	13,274 (10%)
2005/2006	14,071 (10.7%)
2006/2007	14,618 (11.2%)
2007/2008	15,274 (11.6%)
2008/2009	16,979 (13%)
All	131,509 (100%)
	
*Principal condition:*	
Embedded/impacted teeth	64,327 (48.9%)
Dental caries	11,865 (9.0%)
Disorders teeth andsupporting structures	11,136 (8.5%)
Other fractures	8,149 (6.2%)
Malignant neoplasms	5,191 (3.9%)
Pulp/periapical conditions	4,431 (3.4)
Other diseases of the jaw	4,156 (3.2%)
Jaw fractures	3,988 (3.0%)
Dentofacial anomalies	3,439 (2.6%)
Gingivitis and periodontitis	3,080 (2.3%)
All other conditions	11,747 (8.9%)
	
*Insurance status:*	
Private insurance	83,193 (63.3%)
No insurance	48,316 (37.7%)
	
*IRSD*	
Quintile 1 (most disadvantaged)	8,559 (6.5%)
Quintile 2	16,541 (12.6%)
Quintile 3	14,538 (11.1%)
Quintile 4	29,599 (22.5%)
Quintile 5 (least disadvantaged)	61,666 (46.9%)
All	131,509 (100%)

**Table 3:** Hospitalisation rates over 10 years by IRSD quintile

*IRSD*	*Rate*	*95% CI*
Quintile 1 (most disadvantaged)	1,002.22	979.49, 1,025.55
Quintile 2	723.77	711.91, 735.83
Quintile 3	692.49	680.39, 704.81
Quintile 4	741.70	732.60, 750.93
Quintile 5 (least disadvantaged)	964.14	955.94, 972.43

Abbreviations: CI, confidence interval; IRSD, Index of Relative Socioeconomic Disadvantage.

*Rates are per 100,000 persons and adjusted for age and IRSD status.

**Table 4:** Insurance status, length of stay, and cost over 10 years by IRSD quintile:

*IRSD*	*Insured (%)*	*Not-insured (%)*	*Days in hospital mean (s.d.)*	*Direct costs (AU$) mean (s.d.)*
1	2,439 (28.5%)	6,120 (71.5%)	2.07 (3.9)	3642 (7823)
2	9,542 (57.7%)	69,999 (14.6%)	1.57 (3.4)	3242 (6762)
3	7,731 (53.2%)	6,807 (46.8%)	1.74 (3.5)	3513 (7726)
4	17,220 (58.2%)	12,379 (41.8%)	1.51(2.9)	3178 (6653)
5	46,028 (74.6%)	15,637 (25.4%)	1.37 (2.7)	2942 (6372)

IRSD quintile 1=most disadvantaged, quintile 5=least disadvantaged.

Abbreviation: IRSD, Index of Relative Socioeconomic Disadvantage.

**Table 5:** Odds ratios (and 95% confidence intervals) for principal diagnosis at admission, by age group, in relation to socioeconomic disadvantage of area of residence:

	*18–39 years*	*40–59 years*	*60–79 years*	*80+ years*
*Embedded/ impacted teeth*				
Quintile 5 (least deprived)	Reference	Reference	Reference	Reference
Quintile 1 (most deprived)	0.24 (0.23–0.26)*	0.37 (0.32–0.43)*	0.42 (0.28–0.62)*	0.35 (0.08–1.45)
Quintile 2	0.62 (0.59–0.65)*	0.71 (0.64–0.78)*	0.61 (0.49–0.76)*	0.64 (0.31–1.31)
Quintile 3	0.53 (0.51–0.56)*	0.69 (0.63–0.76)*	0.69 (0.56–0.86)*	0.51 (0.26–1.02)
Quintile 4	0.72 (0.69–0.75)*	0.87 (0.81–0.94)*	0.90 (0.75–1.08)	0.68 (0.35–1.30)
				
*Dental caries*				
Quintile 5 (least deprived)	Reference	Reference	Reference	Reference
Quintile 1 (most deprived)	2.65 (2.37–2.95)*	1.13 (1.01–1.27)**	0.78 (0.63–0.97)*	0.72 (0.45–1.16)
Quintile 2	2.12 (1.93–2.33)*	1.27 (0.64–0.78)*	0.88 (0.76–1.01)	0.68 (0.49–0.94)**
Quintile 3	1.84 (1.65–2.04)*	1.07 (0.63–0.76)	0.70 (0.60–0.81)*	0.24 (0.16–0.36)*
Quintile 4	1.68 (1.55–1.82)*	1.09 (0.81–0.94)*	0.69 (0.61–0.79)*	0.70 (0.52–0.95)**
				
*Disorders teeth/ sup structures*				
Quintile 5 (least deprived)	Reference	Reference	Reference	Reference
Quintile 1 (most deprived)	0.46 (0.36–0.57)*	0.23 (0.19–0.27)*	0.16(0.12–0.22)**	0.17 (0.05–0.55)**
Quintile 2	0.80 (0.70–0.92)*	0.49 (0.44–0.54)*	0.40 (0.35–0.46)**	0.67 (0.44–1.03)
Quintile 3	0.98 (0.86–1.12)	0.52 (0.47–0.57)*	0.43 (0.37–0.49)**	0.53 (0.35–0.79)**
Quintile 4	0.73 (0.66–0.81)*	0.47 (0.43–0.51)*	0.45 (0.39–0.51)**	0.41 (0.25–0.66)*
				
*Other fractures:*				
Quintile 5 (least deprived)	Reference	Reference	Reference	Reference
Quintile 1 (most deprived)	4.80 (4.42–5.22)*	3.02 (2.56–3.55)*	2.53 (1.89–3.40)*	1.05 (0.59–1.88)
Quintile 2	1.59 (1.46–1.74)*	1.49 (1.26–1.77)*	1.34 (1.03–1.74)*	1.64 (1.16–2.31)*
Quintile 3	2.01 (1.84–2.20)*	1.59 (1.35–1.87)*	1.23 (0.93–1.62)	0.93 (0.65–1.33)
Quintile 4	1.42 (1.32–1.53)*	1.47 (1.28–1.69)*	1.42 (1.12–1.80)*	1.59 (1.15–2.21)**
				
*Malignancies:*				
Quintile 5 (least deprived)	Reference	Reference	Reference	Reference
Quintile 1 (most deprived)	3.13 (2.12–4.63)*	2.36 (1.01–1.27)*	3.32 (2.60–3.93)*	3.49 (2.35–5.91)*
Quintile 2	1.46 (0.98–2.19)	1.66 (1.16–1.40)*	1.95 (1.70–2.23)*	1.26 (0.90–1.77)
Quintile 3	2.00 (1.34–2.96)*	1.70 (0.97–1.18)*	2.13 (1.86–2.44)*	1.43 (1.07–1.91)**
Quintile 4	2.41 (1.80–3.21)*	1.37 (1.09–1.18)*	2.02 (1.78–2.29)*	1.23 (0.89–1.69)
				
*Pulp, periapical:*				
Quintile 5 (least deprived)	Reference	Reference	Reference	Reference
Quintile 1 (most deprived)	5.20 (4.52–5.97)*	1.86 (1.58–2.20)*	0.90 (0.62–1.29)	0.60 (0.18–1.94)
Quintile 2	2.25 (1.95–2.59)*	1.27 (1.09–1.48)*	0.91 (0.71–1.17)	0.89 (0.46–1.73)
Quintile 3	2.60 (2.24–3.01)*	1.37 (1.18–1.59)*	0.86 (0.66–1.12)	0.64 (0.33–1.24)
Quintile 4	2.04 (1.81–2.31)*	1.13 (0.99–1.29)**	0.82 (0.65–1.04)	1.43 (0.85–2.41)
				
*Other diseases:*				
Quintile 5 (least deprived)	Reference	Reference	Reference	Reference
Quintile 1 (most deprived)	1.58 (1.01–2.49)**	2.75 (2.37–3.19)*	1.69 (1.38–2.07)*	0.64(0.08–4.84)
Quintile 2	0.74 (0.46–1.18)	1.38 (1.19–1.61)*	2.18 (1.90–2.50)*	1.04 (0.34–3.09)
Quintile 3	1.08 (0.69–1.69)	1.45 (1.25–1.68)*	1.76 (1.52–2.04)*	0.71 (0.51–0.92)
Quintile 4	1.12 (0.81–1.54)	1.44 (1.27–1.63)*	1.72(1.50–1.96)*	0.66 (0.19–2.27)
				
*Jaw fractures:*				
Quintile 5 (least deprived)	Reference	Reference	Reference	Reference
Quintile 1 (most deprived)	4.99 (4.48–5.56)*	3.91 (3.09–4.95)*	1.31 (0.70–2.42)	0.56 (0.17–1.81)
Quintile 2	1.74 (1.54–1.95)*	2.05 (1.61–2.60)*	0.71 (0.41–1.23)	1.15 (0.64–2.06)
Quintile 3	1.99 (1.76–2.25)*	1.88 (1.47–2.40)*	0.88 (0.53–1.47)	0.94 (0.54–1.63)
Quintile 4	1.41 (1.27–1.56)*	1.85 (1.51–2.28)*	1.03 (0.67–1.59)	1.26 (0.74–2.14)

Quintiles: Based on IRSD score of area of residence.

Abbreviation: IRSD, Index of Relative Socioeconomic Disadvantage.

*P<0.001, **P<0.05.
